# Paradoxical response of intracranial pressure to shunt valve setting adjustments

**DOI:** 10.1007/s00701-020-04462-y

**Published:** 2020-06-24

**Authors:** Linda D’Antona, Claudia Louise Craven, Melida Andrea Jaime Merchan, Simon David Thompson, Fion Bremner, Lewis Thorne, Manjit Singh Matharu, Laurence Dale Watkins, Ahmed Kassem Toma

**Affiliations:** 1 Victor Horsley Department of Neurosurgery, National Hospital for Neurology and Neurosurgery, Queen Square, WC1N 3BG, London, UK; 2 UCL Queen Square Institute of Neurology, Queen Square, WC1N 3BG, London, UK; 3 Department of Neuro-Ophthalmology, National Hospital for Neurology and Neurosurgery, London, UK; 4 Headache and Facial Pain Group, National Hospital for Neurology and Neurosurgery, London, UK

**Keywords:** Adjustable valve, Cerebrospinal fluid, Intracranial pressure, Intracranial pressure monitoring, Ventriculoperitoneal shunt

## Abstract

**Background:**

The hydrodynamics of cerebrospinal fluid shunts have been described in vitro; however, knowledge on the response of intracranial pressure (ICP) to valve settings adjustments in vivo is limited. This study describes the effect of adjusting the shunt valve setting on ICP in a cohort of patients with complex symptom management.

**Method:**

Single-centre retrospective observational study. Patients who underwent ICP-guided valve setting adjustments during 24-h continuous ICP monitoring, between 2014 and 2019, were included. Patients with suspected shunt malfunction were excluded. Median night ICP before and after the valve adjustments were compared (Δ night ICP). The responses of ICP to valve adjustment were divided into 3 different groups as follows: expected, paradoxical and no response. The frequency of the paradoxical response and its potential predicting factors were investigated.

**Results:**

Fifty-one patients (37 females, 14 males, mean age 38 years) receiving 94 valve setting adjustments met the study inclusion criteria. Patients’ underlying conditions were most commonly hydrocephalus (47%) or idiopathic intracranial hypertension (43%). The response of ICP to valve setting adjustments was classified as ‘expected’ in 54 cases (57%), ‘paradoxical’ in 17 cases (18%) and ‘no effect’ (Δ night ICP < 1 mmHg) in 23 cases (24%). There was a significant correlation between the Δ night ICP and the magnitude of valve setting change in both the investigated valves (Miethke ProGAV, *p* = 0.01 and Medtronic Strata, *p* = 0.02).

**Conclusions:**

Paradoxical ICP changes can occur after shunt valve setting adjustments. This observation should be taken into account when performing ICP-guided valve adjustments and is highly relevant for the future development of “smart” shunt systems.

## Introduction

The management of chronic hydrocephalus and cerebrospinal fluid (CSF) dynamics disorders is often complex. CSF shunt systems provide the possibility to tailor patients’ treatment thanks to the availability of adjustable differential pressure valves and, more recently, adjustable gravitational components. The creation of “smart” shunts, able to perform automated adjustments has been pursued and envisioned for years [12]. The “smart” shunt is expected to perform automated titration of the CSF flow drainage based on two types of feedback: patients’ symptoms (especially headache) and intracranial pressure (ICP) measurements. However, the interpretation of both headache and ICP in patients with CSF dynamics disorders is challenging. For example, it has been demonstrated that primary headaches are often a comorbidity, making the interpretation of CSF-related headache difficult (e.g. migraine in idiopathic intracranial hypertension) [13]. Additionally, evidence on what can be considered normal ICP and therefore which ICP values should be targeted is limited [3].

Another important obstacle for the creation of “smart’ shunts is that information on the effect of valve adjustments on ICP in vivo is limited. Benchmark laboratory tests are routinely performed to assess shunt systems functionality and they help predict the effect that valve settings adjustments will have on CSF flow and consequently on ICP [1, 6, 8, 10]. However, due to the complexity of the CSF dynamics, these predictions can fail in vivo.

This study describes the effects of adjusting shunt valve settings on ICP in a cohort of adult patients with complex symptom management. The primary objective was to assess the frequency of paradoxical changes in ICP following valve setting adjustments. The secondary objective was to identify potential predictive factors for this event.

## Methods and materials

This is a single-centre retrospective observational study conducted at the National Hospital for Neurology and Neurosurgery (London).

### Patients selection

The clinical ICP monitoring database, which is maintained prospectively, was screened to identify patients who underwent continuous 24-h ICP monitoring between January 2014 and April 2019. Patients who had a shunt valve setting adjustment during the monitoring period and recording of ICP 24 h before and 24 h after the valve adjustment were selected. The main exclusion criteria were as follows: (i) incomplete ICP recording (<24 h) before or after valve setting adjustment, (ii) lack of information about the valve setting adjustment and (iii) suspected shunt blockage/dysfunction addressed with shunt revision surgery in the following 6 months. Additionally, (iv) patients who had valve setting adjustments of gravitational components (e.g. Miethke ProSA) or simultaneous adjustments of more than one shunt component were excluded.

Shunt malfunctions were suspected on the basis of the clinical picture, imaging findings and ICP monitoring results. More specifically, patients who were suspected to have shunt malfunction were excluded from the study in case of imaging evidence of disconnections/misplacements of shunt components, sudden/extreme increase in ventricular size, acute clinical deteriorations and/or shunt reservoir assessment suggesting shunt blockage.

### ICP monitoring

ICP monitoring admissions were conducted according to an established local protocol [7, 14]. Indications for this investigation were discussed in a multidisciplinary context including the opinions of neurosurgeons, neurologists, ophthalmologists and radiologists. Patients selected for this study underwent continuous intraparenchymal ICP monitoring for ICP-guided valve setting adjustments due to their complex symptom management. The 24-h ICP monitoring data were analysed with the software ICM+ (Cambridge Enterprise, United Kingdom) and the results summarized in terms of median ICP and median pulse amplitude during the 24 h, daytime and night-time.

### Data collection

The following information on patients’ baseline characteristics was collected: age, gender, diagnosis, type of shunt (ventriculoperitoneal, lumboperitoneal, other), type of valve, presence of gravitational (or anti siphoning) component, age of shunt (months from insertion or last revision) ventriculomegaly in most recent brain imaging (defined as Evan’s index ≥ 0.3).

Valve setting changes were defined as ‘upwards’ changes when the valve setting was increased and ‘downwards’ changes when the valve setting was decreased. The difference between post-adjustment and pre-adjustment median night ICP was calculated (Δ night ICP). Compared to median 24-h ICP or median daytime ICP, the median night ICP (measured between 00:00 and 06:00 am) is more stable as measured when the patient is supine in bed and not affected by the patient’s level of activity. For this reason, the Δ night ICP was chosen as the marker of ICP change for all statistical analysis.

The effect of the valve setting adjustment on night ICP was defined as ‘expected’, ‘paradoxical’ or ‘no effect’:

- ‘Expected effect’: an ‘upwards’ valve setting adjustment generating an increase in night median ICP (Δ night ICP > 1 mmHg) or a ‘downwards’ valve setting adjustment generating a decrease in night median ICP (Δ night ICP < − 1 mmHg);
- ‘Paradoxical effect’: an ‘upwards’ valve setting adjustment generating a decrease in night median ICP (Δ night ICP < − 1 mmHg) or a ‘downwards’ valve setting adjustment generating an increase in night median ICP (Δ night ICP > 1 mmHg);
- ‘No effect’: a valve setting change with negligible impact on night ICP (≤ 1 mmHg).

### Statistical analysis

Patients’ baseline demographic, clinical characteristics, shunt features and ICP monitoring results were summarized. The proportion of valve adjustments resulting in ‘expected effect’, ‘paradoxical effect’ and ‘no effect’ on median night ICP was calculated. Continuous variables were summarized as means (±standard deviation) and categorical variables as percentages.

The difference between the three effect groups (‘expected’, ‘paradoxical’ and ‘no effect’) was tested for the following variables: age, gender, diagnosis (hydrocephalus, idiopathic intracranial hypertension, other), ventricular size (small, large), shunt type (ventriculoperitoneal, lumboperitoneal, other), shunt age, valve type (Miethke ProGAV, Medtronic Strata, other), type of valve setting adjustment (upwards, downwards), baseline median ICP and baseline median pulse amplitude. When available, historical ICP monitoring results preceding the shunt insertion (or the latest revision) were compared with the baseline pre-adjustment ICP monitoring results to provide further evidence of the correct functionality of the shunt system (“Historical” Δ night ICP = pre-adjustment median night ICP minus pre-shunt median night ICP).

Fisher’s exact test was used to compare categorical variables. Depending on the normality of distribution, Kruskal-Wallis H test or one-way analysis of variance (ANOVA) were used to compare continuous variables.

For valve types with more than 10 observations, a linear regression analysis was performed to assess the relationship (linearity) between Δ night ICP in mmHg (dependent variable) and magnitude of valve setting adjustment (post-adjustment setting minus baseline setting, independent variable).

Statistical significance level of 0.05 was used. Microsoft® Excel for Mac (version 16.25), Stata© (version 15.0) and GraphPad Prism (version 8.3.1) were used for the data collection, statistical analysis and graphs creation.

## Results

**Fig. 1:**
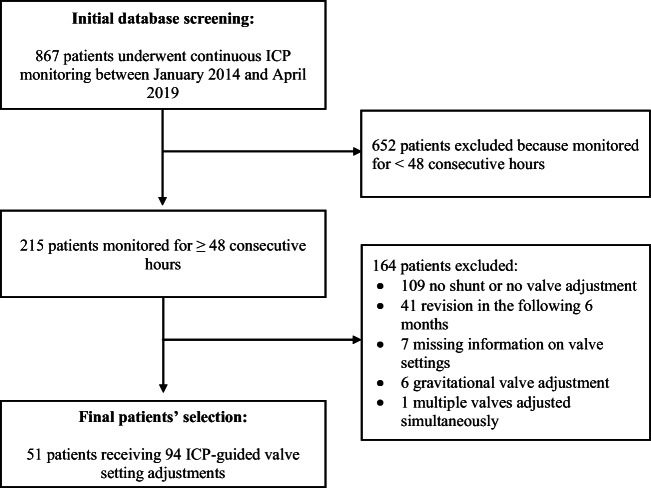
Flow diagram of patients’ selection process and reasons for exclusion

Between January 2014 and April 2019, 867 patients underwent continuous 24-h ICP monitoring. Figure 1 shows the patients selection process and reasons for exclusion. Fifty-one patients receiving 94 valve setting adjustments met the study inclusion criteria. The population demographic characteristics, diagnosis, shunt features and baseline ICP monitor results are described in Table 1.

**Table 1:** Population baseline characteristics

Sex	*N* (%)
- Female - Male	37 (73) 14 (27)
Age (years)	Mean (SD)
	38 (14)
Diagnosis	*N* (%)
- Hydrocephalus - Idiopathic intracranial hypertension - Chiari malformation - LOVA	24 (47) 22 (43) 3 (6) 2 (4)
Shunt type	*N* (%)
- Ventriculoperitoneal - Lumboperitoneal - Ventriculoatrial - Ventriculopleural - Ventriculoperitoneal and lumboperitoneal - Ventriculoperitoneal and lumbopleural	34 (67) 12 (24) 2 (4) 1 (2) 1 (2) 1 (2)
Shunt age	Mean months (SD)
	32 (39)
Valve type	*N* (%)
- Miethke ProGAV - Medtronic Strata - Codman Certas - Sophysa Polaris - Codman Hakim Adjustable - Multiple valves	23 (45) 19 (37) 4 (8) 3 (6) 1 (2) 1 (2)
Ventricles size	N (%)
- Small	43 (84)
- Large	8 (16)
Baseline median ICP (mmHg)	Mean of the medians (SD)
- 24-h - Night	3 (5) 6 (6)
Baseline median pulse amplitude (mmHg)	Mean of the medians (SD)
- 24-h - Night	5 (2) 4 (2)

*ICP* intracranial pressure

*LOVA* longstanding overt ventriculomegaly in adults

*SD* standard deviation

**Fig. 2:**
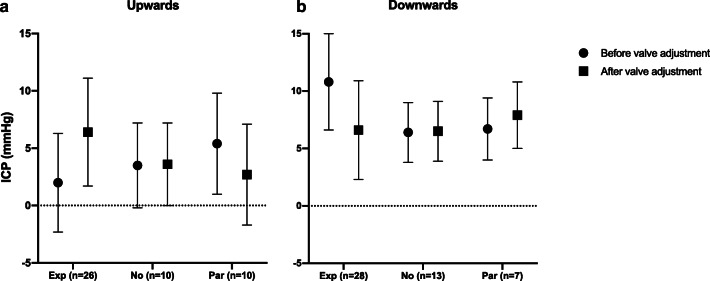
Summary of pre- and post-shunt adjustment night ICP (mean and SD) stratified by direction of valve setting adjustment (upwards/downwards) and effect of valve adjustment on ICP (Exp, expected effect; No, no effect; Par, paradoxical effect)

Valve setting adjustments resulted in the ‘expected effect’ on median night ICP in 54 cases (57%), ‘no effect’ in 23 cases (24%) and a ‘paradoxical effect’ in 17 cases (18%). Pre- and post-valve adjustment night ICP results stratified by effect group are described in Fig. 2. The baseline demographic, clinical, shunt and ICP characteristics did not significantly differ among the three effect groups (Table 2).

**Table 2:** Comparison of baseline demographic, clinical, shunt and intracranial pressure characteristics between three groups of patients with different responses to valve setting adjustment: ‘expected’, ‘paradoxical’ and ‘no effect’ groups

	‘Expected effect’ group (*n* = 54)^a^	‘No effect’ group (*n* = 23)^b^	‘Paradoxical effect’ group (*n* = 17)^c^	*p* value
Sex, *N* (row %)				
- Female - Male	37 (54) 17 (65)	18 (26) 5 (19)	13 (19) 4 (15)	0.67
Age, Mean (SD)	37 (14)	37 (12)	41 (17)	0.60
Diagnosis, *N* (row %)				
- Hydrocephalus - IIH - Other	29 (62) 23 (53) 3 (60)	9 (19) 12 (28) 2 (40)	9 (19) 8 (19) 0 (0)	0.60
Shunt type, *N* (row %)				
- Lumboperitoneal - Ventriculoperitoneal - Other	9 (56) 35 (58) 10 (56)	5 (31) 11 (18) 7 (39)	2 (13) 14 (23) 1 (6)	0.25
Shunt age in months, Mean (SD)	38 (40)	23 (15)	17 (21)	0.40
Valve type, *N* (row %)				
- Medtronic Strata - Miethke ProGAV - Other	19 (59) 27 (52) 8 (80)	9 (28) 12 (23) 2 (20)	4 (13) 13 (25) 0 (0)	0.32
Ventricles size, *N* (row %)				
- Small - Large	44 (56) 10 (63)	21 (27) 2 (13)	13 (17) 4 (25)	0.52
Baseline median ICP in mmHg, Mean (SD)				
- 24-h - Night	3 (6) 7 (6)	3 (4) 5 (4)	3 (4) 6 (4)	0.86 0.56
Baseline median pulse amplitude in mmHg, Mean (SD)				
- 24-h - Night	4 (2) 4 (2)	5 (1) 5 (2)	5 (2) 4 (2)	0.50 0.69
Valve setting change type, *N* (row %)				
- Downwards - Upwards	28 (58) 26 (57)	13 (27) 10 (22)	7 (15) 10 (22)	0.66
Δ night ICP in mmHg, Mean (SD) Comparison of pre- and post-valve adjustment ICP recordings.	4 (3)	1 (0)	4 (3)	<0.01
“Historical” Δ night ICP^d^ in mmHg, Mean (SD) Information available for 25 patients only (13 ‘expected effect’, 6 ‘no effect’ and 6 ‘paradoxical effect’)	5 (4)	4 (3)	5 (6)	0.40

^a^‘Expected effect’: night ICP change > 1 mmHg in the expected/desired direction

^b^‘No effect’: negligible night ICP change (≤ 1 mmHg)

^c^‘Paradoxical effect’: night ICP change > 1 mmHg in the unexpected/undesired direction

^d^ “Historical” Δ night ICP = baseline pre-shunt median night ICP - pre-adjustment median night ICP

*ICP* intracranial pressure, *IIH* idiopathic intracranial hypertension, *SD* standard deviation

**Fig. 3:**
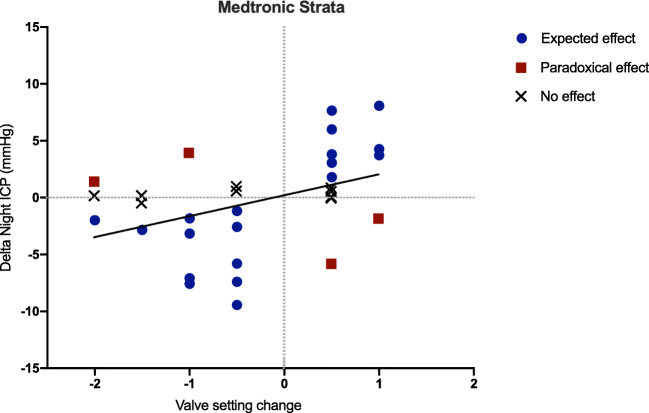
Scatter plot and line of best fit of night intracranial pressure change (mmHg) and magnitude of Medtronic Strata setting adjustment (post-adjustment setting minus baseline setting)

**Fig. 4:**
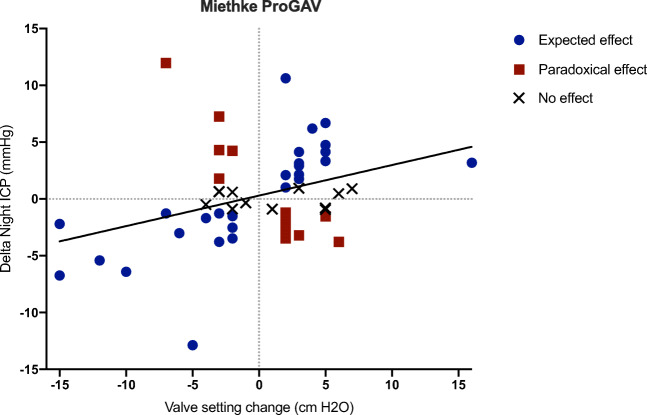
Scatter plot and line of best fit of night intracranial pressure change (mmHg) and magnitude of Miethke ProGAV setting adjustment (post-adjustment setting minus baseline setting)

Medtronic Strata and Miethke ProGAV were the only valves with 10 or more observations. Two unadjusted linear regression models confirmed the association between the magnitude of valve setting adjustment (post-adjustment setting minus baseline setting) and Δ night ICP for both Medtronic Strata (*β* = 1.84; 95% CI 0.30 to 3.38, *P* = 0.02; adjusted *R*^2^ = 0.14) and Miethke ProGAV valves (*β* = 0.27; 95% CI 0.07 to 0.47, *P* = 0.01; adjusted *R*^2^ = 0.11). Figures 3 and 4 display the night ICP change caused by different magnitudes of valve setting adjustments for Medtronic Strata and Miethke ProGAV respectively.

## Discussion

This single-centre retrospective observational study describes the effects of 94 shunt valve setting adjustments on ICP in a cohort of patients investigated with continuous 24-h ICP monitoring. To the best of our knowledge, this is the first description of paradoxical ICP changes following valve setting adjustments of functional shunts in a large cohort of patients. Among 94 valve setting adjustments, paradoxical ICP changes were observed in 18% of the cases, and no significant change in ICP (Δ night ICP < 1 mmHg) in 24%. This observation should be taken into account when performing ICP-guided valve setting adjustments and suggests that a paradoxical response to valve setting adjustments does not always indicate shunt malfunction (only patients with functioning shunts were included in this study). This is further proof of the complexity of CSF drainage shunts in the context of chronic hydrocephalus and other CSF dynamics disorders.

The baseline characteristics of the three effect groups (‘expected’, ‘paradoxical’ and ‘no effect’) did not significantly differ (Table 2), making the identification of potential mechanisms for the paradoxical response difficult. It could be hypothesized that in patients with small ventricles (e.g. IIH), ‘downwards’ valve setting adjustments could cause obstruction of the ventricular catheter lumen secondary to a collapse of the ventricular walls. However, our results did not show any association between paradoxical response and small ventricles or IIH. On the other hand, ‘upwards’ valve setting adjustments could displace intraluminal debris from the ventricular catheter and cause a paradoxical response. While we would expect older shunts to present such intraluminal debris, a significant association between shunt age and paradoxical effect was not found in this study. Previous experience suggests that, if there are no in-between interventions, different sets of elective 24-h ICP monitoring measured in the same patient do not significantly differ [14]. However, the possibility that random ICP changes may have affected the before and/or after valve adjustment ICP results generating a paradoxical ICP response exists. Another hypothesis is that CSF dynamics may require longer times (> 24 h) to adapt to a new valve setting.

The hydrodynamics of valve setting adjustments have been investigated in several in vitro studies [1, 6, 8, 10]. Information on the effect of valve setting adjustments on ICP in vivo is scarce. Farahmand et al. looked at the impact of Medtronic Strata valve adjustments on ICP in a group of 15 patients with communicating hydrocephalus concluding that the ICP change between different valve settings was smaller than the difference previously observed in vitro [11]. Antes et al. found that the patients who do not benefit from ICP-guided adjustments are those who do not demonstrate a significant ICP change (follow-up compared with baseline ICP) [4]. These findings corroborate our observation that 24% of valve setting adjustments generated a minimal ICP change, not exceeding 1 mmHg (‘no effect’ group). Similarly, Bergsneider et al. demonstrated that ICP at different Codman Hakim programmable valve settings were lower than predicted with a linear mathematical model in 11 normal pressure hydrocephalus patients [5]. The previously published studies have an important limitation: They measured ICP for a short period time (minutes) or shortly after a postural change, possibly before the achievement of the ICP ‘steady state’ [4, 5, 11]. To overcome this limitation, we have used median night ICP; this value is obtained from a 6-h continuous measurement recorded at standard times (00:00–06:00) when the patient is expected to be supine in bed. In 2008, Eide and Sorteberg used a similar methodology to describe the effect of valve setting adjustments in the ICP of 2 normal pressure hydrocephalus patients [9]. Interestingly, their data shows one episode of ICP increase after a downward valve setting adjustment in a patient with functional shunt; this is in keeping with what we define as a ‘paradoxical effect’.

The main limitation of our study is the retrospective analysis of prospectively collected data; however, the standardized approach for ICP monitoring data recording, analysis and storage might have reduced the disadvantages of this design. Body position has known effects on ICP [2], different postures in the pre- and post-valve adjustment readings could have affected our results. This is the reason why we used the median night ICP, which is recorded between 00:00 and 06:00 am when the patients were supine. Technical issues with ICP monitoring could have occurred, but they are unlikely since a routine raw ICP data quality assessment was performed before each 24-h ICP monitoring analysis. The lack of detailed information on the patients’ symptoms and their changes in relation to the valve setting adjustments as well as intra-abdominal pressure measurements are further shortcomings. Despite being the largest study on this topic, a larger cohort of patients would have allowed a better understanding of potential predictors of the response to valve adjustments. Moreover, the possibility that some of the shunts in the ‘no/paradoxical effect’ groups were non-functional cannot be excluded with absolute certainty. Nevertheless, we did not find any clinical evidence of malfunctioning (imaging and reservoir assessment) and there was no statistical difference among groups in any of the tested variables including the “historical” Δ night ICP. Finally, there is a selection bias due to fact that only patients with complex symptom management were included in this study and this aspect should be taken into account when interpreting these results.

Future prospective studies employing implantable telemetric ICP monitoring devices such as Sensor Reservoir (Miethke) and Neurovent-P-Tel (Raumedic) might be able to assess the effect of valve settings adjustments on ICP for longer periods of time and clarify predicting factors for ‘paradoxical’ ICP response to adjustment. Due to the limited number of cases, it was not possible to describe the effect of gravitational valve adjustments on ICP and this aspect should also be addressed by future research. A better understanding of the dynamics of CSF shunts in vivo is essential for the future development of smart shunts able to generate automated responses to patients’ symptoms and ICP.

Paradoxical ICP changes can occur in the 24 h following shunt valve setting adjustments. This observation should be taken into account when performing ICP-guided valve adjustments and is highly relevant for the future development of “smart” shunt systems.

## Abbreviations

CSF: Cerebrospinal fluid
ICP: Intracranial pressure
IIH: Idiopathic intracranial hypertension
